# Aberrant BUB1 Overexpression Promotes Mitotic Segregation Errors and Chromosomal Instability in Multiple Myeloma

**DOI:** 10.3390/cancers12082206

**Published:** 2020-08-06

**Authors:** Yuto Fujibayashi, Reiko Isa, Daichi Nishiyama, Natsumi Sakamoto-Inada, Norichika Kawasumi, Junko Yamaguchi, Saeko Kuwahara-Ota, Yayoi Matsumura-Kimoto, Taku Tsukamoto, Yoshiaki Chinen, Yuji Shimura, Tsutomu Kobayashi, Shigeo Horiike, Masafumi Taniwaki, Hiroshi Handa, Junya Kuroda

**Affiliations:** 1 Division of Hematology and Oncology, Department of Medicine, Kyoto Prefectural University of Medicine, Kyoto 602-8566, Japan; f-yuto@koto.kpu-m.ac.jp (Y.F.); isa-r@koto.kpu-m.ac.jp (R.I.); daichi@koto.kpu-m.ac.jp (D.N.); n-saka@koto.kpu-m.ac.jp (N.S.-I.); huntail816@icloud.com (N.K.); junko-y@koto.kpu-m.ac.jp (J.Y.); s-kuwaha@koto.kpu-m.ac.jp (S.K.-O.); m-yayoi@koto.kpu-m.ac.jp (Y.M.-K.); ttsuka@koto.kpu-m.ac.jp (T.T.); y-chinen@koto.kpu-m.ac.jp (Y.C.); yshimura@koto.kpu-m.ac.jp (Y.S.); t-koba@koto.kpu-m.ac.jp (T.K.); shoriike@koto.kpu-m.ac.jp (S.H.); taniwaki@koto.kpu-m.ac.jp (M.T.); 2 Department of Hematology, Fukuchiyama City Hospital, Kyoto 620-8505, Japan; 3 Center for Molecular Diagnostics and Therapeutics, Kyoto Prefectural University of Medicine, Kyoto 602-8566, Japan; 4 Department of Hematology, Gunma University Graduate School of Medicine, Gunma 371-8511, Japan; handahiroshi@gunma-u.ac.jp

**Keywords:** multiple myeloma, BUB1, chromosome segregation error, chromosomal instability, clonogenicity

## Abstract

Chromosome instability (CIN), the hallmarks of cancer, reflects ongoing chromosomal changes caused by chromosome segregation errors and results in whole chromosomal or segmental aneuploidy. In multiple myeloma (MM), CIN contributes to the acquisition of tumor heterogeneity, and thereby, to disease progression, drug resistance, and eventual treatment failure; however, the underlying mechanism of CIN in MM remains unclear. Faithful chromosomal segregation is tightly regulated by a series of mitotic checkpoint proteins, such as budding uninhibited by benzimidazoles 1 (BUB1). In this study, we found that BUB1 was overexpressed in patient-derived myeloma cells, and BUB1 expression was significantly higher in patients in an advanced stage compared to those in an early stage. This suggested the involvement of aberrant BUB1 overexpression in disease progression. In human myeloma-derived cell lines (HMCLs), BUB1 knockdown reduced the frequency of chromosome segregation errors in mitotic cells. In line with this, partial knockdown of BUB1 showed reduced variations in chromosome number compared to parent cells in HMCLs. Finally, BUB1 overexpression was found to promote the clonogenic potency of HMCLs. Collectively, these results suggested that enhanced BUB1 expression caused an increase in mitotic segregation errors and the resultant emergence of subclones with altered chromosome numbers and, thus, was involved in CIN in MM.

## 1. Introduction

Multiple myeloma (MM) is a cytogenetically and molecularly heterogeneous hematological malignancy that originates from plasma cells and remains mostly incurable, despite recent therapeutic advances. MM is invariably preceded by the precursor state of monoclonal gammopathy of undetermined significance (MGUS), which progresses to smoldering MM and later to MM. Therefore, MM provides an excellent model through which understanding of the multistep evolutional process of cancer progression might be achieved. Early events include acquisition of hyperdiploidy commonly characterized by trisomy of certain odd-numbered chromosomes 3, 5, 7, 9, 11, 15, 19, 21, or immunoglobulin heavy (IGH) chain gene chromosomal translocations. Subsequent to the primary event, numerous types of secondary cytogenetic or molecular aberrations, including chromosomal translocations, copy number abnormalities, somatic mutations, and epigenetic alterations, emerge in an overlapping manner in myeloma cells. This multistep acquisition of a series of genetic abnormalities gives rise to subclones with a selective advantage for disease progression, even under therapeutic pressure [1,2,3,4,5,6]. The involvement of apolipoprotein B mRNA editing enzyme, catalytic polypeptide-like (APOBEC) family enzymes in this process has been shown with the acquisition of genetic mutations [7,8], while lysine demethylase and multiple myeloma SET domain (MMSET) cause epigenetic abnormalities in MM [9]. However, the mechanisms underlying the acquisition of additional chromosome abnormality remain obscure.

Faithful segregation of replicated chromosomes during mitosis is essential for the maintenance of genomic integrity and, therefore, is tightly regulated by the mitotic checkpoint (spindle checkpoint, or spindle assembly checkpoint) mechanism, which requires the coordinated functions of a series of mitotic checkpoint proteins, such as budding uninhibited by benzimidazoles 1 (BUB1), BUB3, BUBR1, mitotic arrest deficient 1 (MAD1), MAD2, and monopolar spindle 1 (MPS1) [10,11]. These checkpoint proteins are recruited to kinetochores unattached to microtubules in metaphase cells and inhibit anaphase-promoting complex/cyclosome, an E3 ubiquitin ligase that drives cells to enter into anaphase, until each chromosome is properly attached to the spindle. After all chromosome pairs are properly attached to the microtubules and aligned, mitotic checkpoint proteins dissociate from anaphase-promoting complex/cyclosome, thus triggering chromosome segregation [12]. Failure of this process causes the acquisition of chromosomal abnormalities and potentially leads to karyotypic evolution in cancer [10].

The serine/threonine kinase BUB1 is a highly conserved multifunctional protein that is essential for the mitotic spindle checkpoint and correct kinetochore-microtubule attachments [13]. In addition, BUB1 regulates targeting of cohesion protein Shugoshin 1 to the centromere through histone H2A phosphorylation and prevents the dissociation of cohesion from centromeres [14]. Thus, BUB1 inactivation has been shown to cause both loss of the spindle checkpoint and severe chromosome segregation defects in non-malignant cells [15]. As for other mitotic checkpoint proteins, BUB1 abnormality is associated with various types of cancers. BUB1 mutations are only occasionally found in cancer [16,17,18], but BUB1 overexpression is a frequent phenomenon that is associated with high proliferative activity of tumor cells and a poor clinical outcome in various solid cancers [18,19,20,21,22,23,24,25,26]. However, the functional involvement of BUB1 in the pathophysiology of MM is unknown. Because multistep chromosomal evolution is the key process in the development and progression of MM, this study was performed to assess the clinical and functional importance of BUB1 in MM.

## 2. Results

### 2.1. BUB1 Overexpression in Patient-Derived Primary Myeloma Cells and Human Myeloma-Derived Cell Lines (HMCLs)

**Figure 1:**
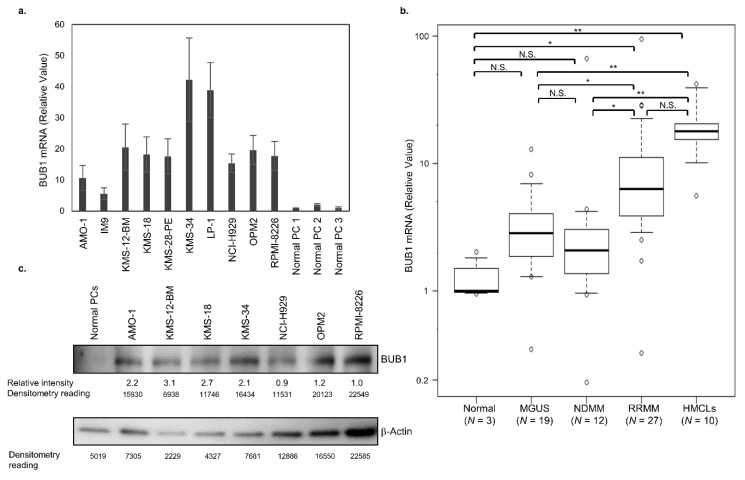
BUB1 is highly expressed in advanced multiple myeloma cells. (**a**) BUB1 transcriptional level in human myeloma-derived cell lines (HMCLs). Normal plasma cells (PCs) were obtained from the bone marrow of 3 healthy donors (PC1-3) and used as the control. Data in the graph are means ± SD of four independent experiments. (**b**) BUB1 transcriptional level in normal PCs, CD138-positive myeloma cells from 39 multiple myeloma (MM) and 19 monoclonal gammopathy of undetermined significance (MGUS) cases, and 10 HMCLs. * *p* < 0.05, ** *p* < 0.01, N.S.: not significant, by one-way ANOVA with a Tukey multiple comparison test. The mRNA expression levels of BUB1 were normalized to those of β-actin in each sample, and their relative values were calculated considering the mean BUB1 transcription levels in normal PCs to be 1.0. (**c**) Immunoblot of BUB1 in HMCLs and normal PCs. The expression level of BUB1 relative to that of β-actin in each HMCL was measured by the densitometric analysis.

BUB1 expression was first examined in 10 HMCLs and normal plasma cells. Compared to normal plasma cells, HMCLs expressed significantly higher BUB1 at the mRNA (Figure 1a,b) and protein (Figure 1c, Figure S1) levels. Next, cluster of differentiation (CD)138-positive plasma cells were isolated, as previously described [27], and BUB1 mRNA in CD138-positive plasma cells from healthy donors (*n* = 3), patients with MGUS (*n* = 19), newly diagnosed MM (NDMM) (*n* = 12), and relapsed/refractory MM (RRMM) (*n* = 27) was examined to investigate its relationship with disease status. BUB1 mRNA tended to be higher in CD138-positive myeloma cells from MGUS and NDMM compared to normal plasma cells, but the difference was not significant. However, the BUB1 mRNA levels in myeloma cells from patients in an advanced stage RRMM and HMCLs were significantly higher than that in normal plasma cells and higher than that in myeloma cells from patients with early-stage disease (MGUS and NDMM). The BUB1 mRNA level did not differ significantly between MGUS and NDMM (Figure 1b). Collectively, these results indicated that myeloma cells expressed an aberrantly higher level of BUB1, which increased with disease progression and might be associated with transformation and immortalization.

### 2.2. Generation of BUB1-Reduced HMCLs

**Figure 2:**
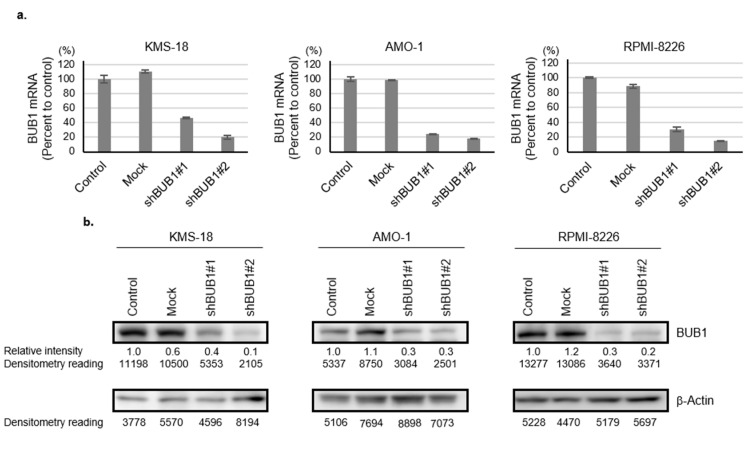
Generation of BUB1-reduced HMCLs. (**a**,**b**) KMS-18, AMO-1, and RPMI-8226 cells were transduced with mock, shBUB1#1, or shBUB1#2. BUB1 mRNA expression (Y-axis: % of control) was determined by qRT-PCR (**a**) and Western blotting (**b**) after puromycin selection. The expression level of BUB1 relative to that of β-actin in each HMCL was measured by densitometric analysis; the BUB1 to the β-actin ratio in controls was defined as 1.0. The protein level relative to the control is shown below each band.

To investigate the biologic and cellular functions of BUB1 overexpression in MM, we tried to reduce BUB1 protein levels close to those in normal plasma cells in three HMCLs—KMS-18, AMO-1, and RPMI-8226 cells—using RNAi technique. These three HMCLs were selected as representatives of the 10 HMCLs examined in the study based on their different protein expression levels of BUB1. Namely, KMS-18 cells, AMO-1 cells, and RPMI-8226 cells expressed the second-highest, the third-highest (intermediate), and the second-lowest BUB1 protein among seven HMCLs examined, respectively. We excluded KMS-12-BM with the highest and NCI-H929 with the lowest BUB1 protein to avoid extreme situations (Figure 1a,c). BUB1 knockdown was achieved by both shBUB1#1 and shBUB1#2, but more potently by shBUB1#2 to <30% for both mRNA and protein levels compared to control and mock cells in the three HMCLs (Figure 2a,b, Figure S2).

### 2.3. No Association of BUB1 Expression with Short-Term Proliferative Potency in HMCLs

**Figure 3:**
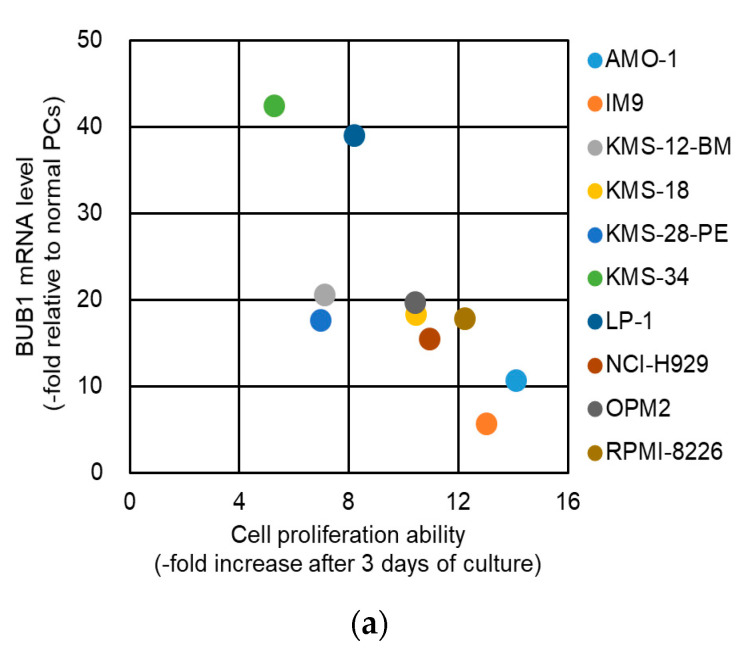

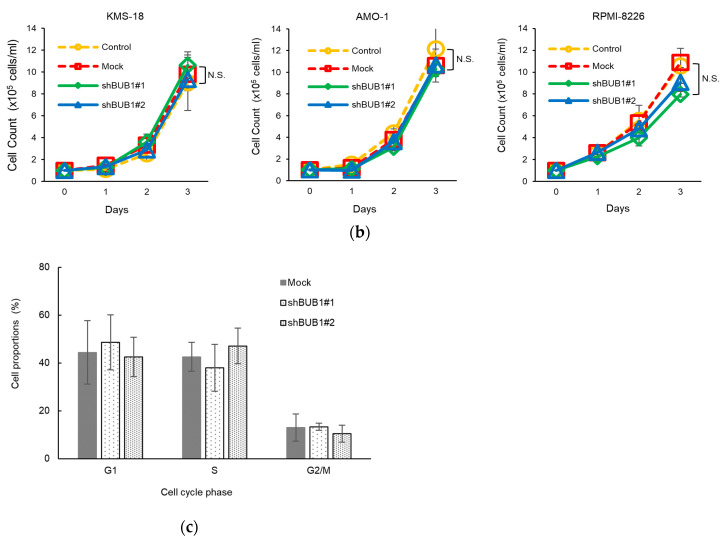
BUB1 expression did not affect the short-term proliferation of HMCLs. (**a**) Association between cell proliferation rates and BUB1 mRNA levels in HMCLs. Fold changes in cell numbers over three days of culture are plotted on the horizontal axis, and BUB1 mRNA levels relative to those in normal plasma cells (PCs) are on the vertical axis. Ten HMCLs were examined. (**b**) Cells were seeded at 1 × 10^5^ cells/mL and grown in the complete liquid culture medium. Cell number was measured by Trypan blue dye exclusion assay over time. Dotted lines show growth of control (yellow circle) and mock-treated (red square) cells, and solid lines show BUB1-reduced cells using shBUB1#1 (green diamonds) and shBUB1#2 (blue triangles). Assays were performed in triplicate, and cell counts are expressed as means ± SD. N.S.: not significant. (**c**) BUB1 knockdown had no effect on cell cycle distribution in HMCLs. Data shown are the representative results with KMS-18 cells. The percentages of cell populations in the G1, S, and G2/M phases of the cell cycle are shown. Data are means ± SD from three independent experiments.

The oncogenic function of BUB1 in cell proliferation is unclear among cancer types [26,28,29,30,31]. We first examined the BUB1 function in the short-term proliferative activity of myeloma cells. As shown in Figure 3a and Figure S3, there was no correlation between the BUB1 mRNA/protein levels and this ability of HMCLs in a complete liquid culture medium, while normal plasma cells did not proliferate in the short-term liquid culture (Author 1, Y.F. unpublished work). There was also no significant impact of BUB1 reduction on proliferation for up to 72 h in liquid culture for the three HMCLs examined (Figure 3b). BUB1 knockdown also had little impact on the cell cycle distribution in HMCLs (Figure 3c). Collectively, these results suggested that BUB1 overexpression did not promote short-term cell proliferative activity in MM.

### 2.4. BUB1 Overexpression Promotes Chromosome Segregation Defects and Chromosome Instability (CIN) in Myeloma Cells

We next investigated the functional involvement of BUB1 overexpression in the acquisition of chromosomal abnormality in MM. For this purpose, we examined the impact of BUB1 knockdown on anaphase chromosome segregation errors in KMS-18 and AMO-1 cells that possess highly complex chromosomal abnormalities, including both numerical and structural abnormalities. In addition, chromosomal features were highly variable among individual metaphase spreads in both HMCLs. In brief, most of the KMS-18 cells harbored more than 60 chromosomes and showed various abnormalities, including chromosome (chr) 1q gain, chr13 deletion, t(4;14) for IGH/MMSET, and t(8;14) for IGH/MYC, while most of the AMO-1 cells possessed more than 80 chromosomes and abnormalities, such as chr1q gain and t(8;14) [32]. Thus, two representative HMCLs utilized in this assay were unstable in terms of chromosomal integrity.

**Figure 4:**
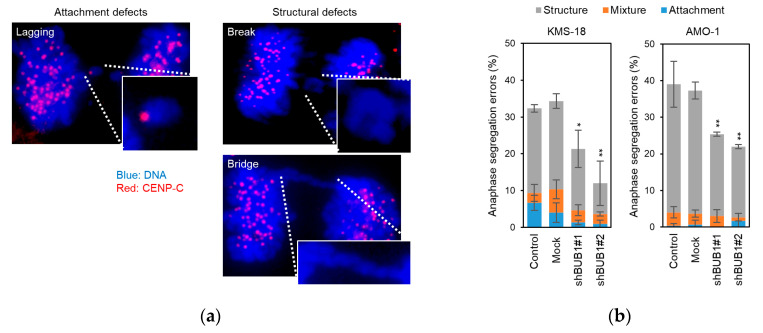
Involvement of BUB1 in chromosome segregation errors in myeloma cells. (**a**) Two types of anaphase chromosome segregation errors were defined: attachment defects, such as a lagging chromosome with a centromere (left), and structural defects (right), such as a chromosome break (right upper) and a chromosomal bridge (right lower). Cells were stained for centromeres with anti- centromere protein (CENP)-C antibody (Ab) with a secondary Ab conjugated with Alexa Fluor 546 (red), and for DNA using DAPI (blue). (× 300) (**b**) Percentage of anaphase cells with chromosome segregation errors in parental, mock-treated, and BUB1-reduced (transfected with shBUB1#1 or shBUB1#2) KMS-18 and AMO-1 cells. A total of 100 anaphase cells were counted in each experiment. Data are means ± SD from three independent experiments. * *p* < 0.05, ** *p* < 0.01 vs. control cells in a two-tailed *t*-test. Gray, blue, and orange bars show the ratios of cells with attachment errors, structural errors, and concomitant attachment and structural errors, respectively.

Chromosome segregation errors were classified into two categories: attachment defects, such as lagging chromosomes with centromeres, and structural defects, including acentric chromosomes and chromosome bridges (Figure 4a). Approximately 30–40% of anaphase cells were found to possess chromosome segregation errors (i.e., attachment and/or structural defects in parental and mock KMS-18 and AMO-1 cells), and BUB1 knockdown significantly decreased the frequency of segregation errors at anaphase in both HMCLs (Figure 4b).

**Figure 5:**
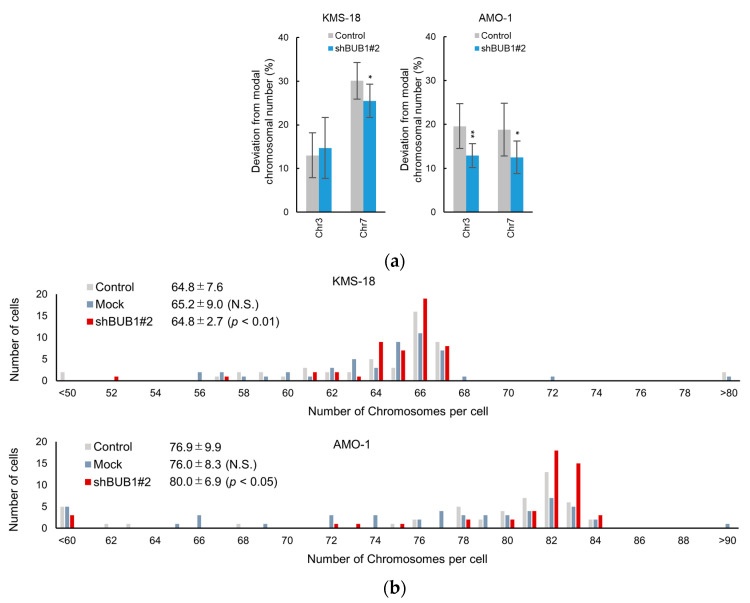
Involvement of BUB1 in the emergence of aneuploidy in myeloma cells. (**a**) Percentage deviations from the modal chromosome number for chromosome (chr) 3 and chr 7 in 10 clones of KMS-18 and AMO-1 cells. A total of 100 interphase cells were evaluated for the numbers of the chr 3 and chr 7 in each clone. Gray and white bars represent parental and BUB1-knockdown cells, respectively. Means ± SD of ten independent clones are shown. * *p* < 0.05, ** *p* < 0.01 by two-tailed *t*-test. (**b**) Distribution of whole chromosome numbers of 50 metaphase cells of control (gray), mock-transduced (blue), and BUB1-reduced (red) KMS-18 (upper) and AMO-1 (lower) cells. Numbers are the average chromosome number, SD, and *p* values vs. control cells in an F test.

Given that the BUB1 expression level determined the frequency of anaphase chromosome segregation errors, we next examined whether the reduction of these errors by BUB1 partial knockdown resulted in a change in the number of chromosomes in a single cell. For this purpose, KMS-18 and AMO-1 cells were subjected to limiting dilution in order to select 10 single-cell clones, which were cultured until the single-cell expanded to 5 million cells (a division of 20 times or more). The numbers of chr3 and chr7 were then counted as representatives of all chromosomes by FISH analysis [33,34] in 100 interphase cells per clone for 10 clones of parental and BUB1-reduced (shBUB1#2) KMS 18 cells and AMO-1 cells to determine the modal chromosome number of the main clone in these cells. The derivative ratio (i.e., cells with chromosome numbers that differed from the modal number) was then determined. While the modal number of chr3 was two in parental KMS-18 cells, these numbers were four in chr3 in AMO-1 cells and four in chr7 in both KMS-18 cells and AMO-1 cells. As a result, the deviations of chromosome numbers from the modal number, i.e., both further increase and decrease of chr3 and chr7, were observed in both HMCLs. As shown in Figure 5a, in parental KMS-18 cells, the derivative ratio was approximately 10% for the number of chr3, which was significantly lower than the ratio for chr7, suggesting that a diploid chromosome is more stable than an aneuploid chromosome. In parental AMO-1 cells, the derivative ratios were around 20% for ch3 and chr7, suggesting largely equivalent instability of the two chromosomes. Importantly, while BUB1 knockdown did not significantly influence the ratio of derivative clones for chr3 in KMS-18 cells, it significantly reduced the rates of deviations from the modal chromosome numbers for chr7 in KMS-18 cells and chr3 and chr7 in AMO-1 cells (Figure 5a, Figure S4).

To evaluate the impact of BUB1 knockdown on total chromosome numbers in more detail, we performed G-banding to count total chromosome numbers, including supernumerary chromosomes and fragments, in 50 mitotic cells of parental and BUB1-reduced KMS-18 and AMO-1 cells. The average total chromosome numbers were not significantly altered by BUB1 knockdown, but standard deviations were significantly reduced by BUB1 reduction in both HMCLs (Figure 5b). Collectively, these results suggested that BUB1 overexpression promoted CIN by increasing chromosome segregation errors, which led to at least a transient increase of subclones harboring different total chromosome numbers from the original clone in HMCLs.

### 2.5. BUB1 Knockdown Significantly Reduces Colony Formation of Myeloma Cells

**Figure 6:**
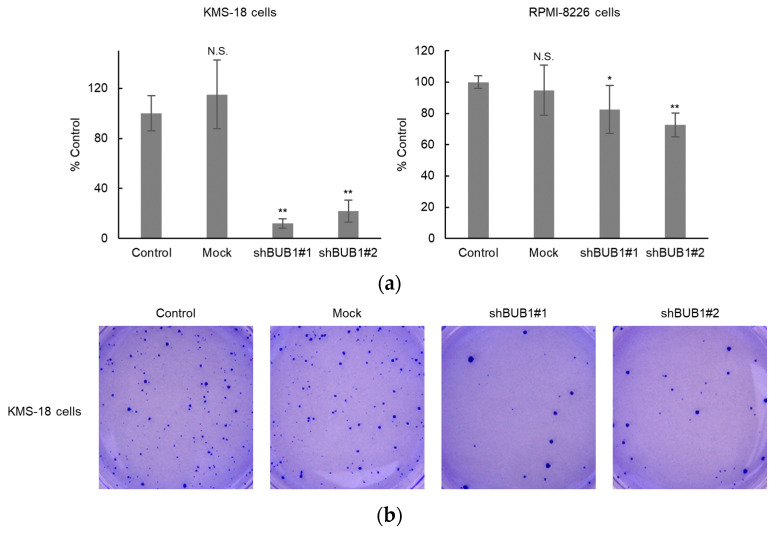
BUB1 knockdown impaired colony-forming capacity in KMS-18 and RPMI-8226 cells. (**a**,**b**) Control, mock-treated, or BUB1-reduced KMS-18 (left) and RPMI-8226 (right) cells were subjected to a colony-forming assay. Triplicate assays were performed in two independent experiments. Data are shown as means ± SD. * *p* < 0.05, ** *p* < 0.01 vs. control cells in a two-tailed *t*-test. N.S.: not significant.

In breast cancer cells, the BUB1 knockdown does not affect short-term cell growth but does impair clonogenic potency [31]. This provoked us to investigate the functional involvement of BUB1 in clonogenic potency in myeloma cells. Evaluation of colony-forming ability using semi-solid methylcellulose-based media as a scaffold showed that parental KMS-18 and RPMI-8226 cells, but not AMO-1 cells, formed well-shaped countable colonies. BUB1 knockdown significantly reduced colony numbers compared to parental and mock KMS-18 and RPMI-8226 cells, with suppression more prominent in KMS-18 cells (Figure 6a,b). Approximately 100 colonies were produced from 2000 KMS-18 cells, but from only 250 RPMI-8226 cells, indicating that KMS-18 cells had lower colony-forming capacity than RPMI-8226 cells. This difference in basal colony-forming capacity might underlie the different degrees of suppression of this capacity by BUB1 reduction in the two HMCLs.

## 3. Discussion

MM remains a hard-to-treat disease despite the advent of new therapeutic strategies in the past two decades. One underlying reason for the difficulty in the development of a curative drug for MM is the lack of a universally targetable molecule due to the high interpatient diversity and the intraclonal heterogeneity of cytogenetic and molecular abnormalities in a single patient [1,2,3,4,5,6,7]. For instance, the type of primary chromosomal abnormality, such as hyperdiploid and immunoglobulin heavy chain gene translocations, determines the cellular characteristics and clinical manifestation and occasionally influences therapeutic decision-making [35,36].

More importantly, additional cytogenetic abnormalities due to CIN occur in subclones of myeloma cells. As in many other cancers [37,38,39,40], an individual chromosome gain and loss may cause overexpression of oncogenes and deletion of tumor suppressors, respectively, and the overlap of these events makes molecular features of myeloma cells more complex and heterogeneous. This eventually gives rise to highly aggressive subclones that expand to extramedullary sites and show high resistance to cytotoxic insults. For example, the presence of subclones harboring additional abnormality of chromosome 1q amplification and/or deletion of chromosome 17p is strongly associated with dismal outcomes [35,36,41,42]. Thus, in the absence of a universal molecular target in the background of high cytogenetic and molecular heterogeneity, clonal evolution is the critical biologic event to be prevented or overcome for improvement of therapeutic outcomes of patients with MM.

Aberrant expression of BUB1 has been repeatedly reported in various cancers [19,20,21,22,23,24,25,26,30,43,44,45]. Because transcription level and protein expression level did not show a clear correlation in our HMCLs examined (Figure 1), the BUB1 protein level might be both positively and negatively regulated by overlapping molecular mechanisms, such as epigenetic regulatory system [46,47], in addition to transcriptional regulation. Interestingly, BUB1 overexpression induces aneuploidy in lymphoma cells through Aurora B kinase (AURKB) hyperactivation [44], while BUB1 downregulation occurs in a subset of acute myeloid leukemia [45]. Given that fine-tuning of the coordinated interplay among checkpoint proteins is essential for faithful chromosome segregation during mitosis, both excessive and insufficient expression of BUB1 could be harmful for prompt chromosome segregation. In this study, we identified aberrant overexpression of BUB1 in patient-derived myeloma cells. Because BUB1 expression was higher in myeloma cells from patients with advanced disease and in HMCLs, BUB1 overexpression might be more associated with disease aggressiveness that is usually accompanied by treatment resistance. We also showed that BUB1 overexpression promoted clonogenic potency in myeloma cells, as well as CIN, through an increase of various types of mitotic chromosomal segregation errors. The simultaneous acquisition of these two biologic characteristics is important in the understanding of myeloma pathophysiology because this combination may promote the establishment of persistently expandable subclones with new additional chromosome abnormalities.

The disease aggressiveness-associated BUB1 overexpression may be translated into the development of a new biomarker for MM. It would be interesting to investigate if the BUB1 expression level may be a surrogate for the degree of CIN in myeloma cells, and thereby, constitute a new prognostic marker. In addition, while we investigated BUB1 expression in CD138-positive patient-derived myeloma cells in this study, it would be also interesting to investigate BUB1 expression in CD138-negative myeloma cells, considering the occasional loss of CD138 expression in myeloma cells of the more advanced clinical phase. However, while AURKB and receptor for hyaluronan-mediated motility (RHAMM) have been suggested to interact with BUB1 to promote cancer stemness and tumor formation [31,44], we were unable to show a functional association between BUB1 overexpression and those two molecules in HMCLs (Author 1. Y.F. unpublished work). Thus, further research is needed to clarify the mechanism underlying BUB1-mediated clonogenicity and tumorigenesis in myeloma cells, and this may also lead to the discovery of a novel therapeutic target, which could be functionally synthetic lethal. In this regard, the role of excess BUB1 expression in the nonhomologous end-joining pathway, an essential mechanism for genome integrity, is the next research topic in association with DNA repair efficiency and treatment resistance in MM [48].

There are some limitations to the study. We showed the involvement of BUB1 in CIN in short-term observation in HMCLs. However, parental KMS-18, AMO-1, and RPMI-8226 cells did not show a change in total chromosome numbers of the modal clone after several weeks with cell division of over 20 times (Figure 5b) and not even after suspension culture and passage for 6 months (Author 1. Y.F. unpublished work), although numerous mitotic chromosomal segregation errors should have emerged during the culture period. For these conflicting results, we proposed two possible explanations. One hypothesis is that BUB1 overexpression as a single molecular abnormality is a prerequisite, but is not sufficient, for the eventual establishment of a subclone with new additional chromosomal abnormalities in MM. In this case, other molecular events, in addition to BUB1 overexpression, need to be identified to fully understand the mechanism of karyotypic evolution. The other hypothesis is that our results were unintentionally influenced by study design, i.e., the use of HMCLs. Basically, aneuploidy is detrimental to a cell and generally confer a fitness disadvantage in physiologic conditions, presumably because gains or losses of whole or large parts of chromosomes result in dosage changes of several hundreds of genes in a cell at the same time, thereby leading to undesirable sudden imbalances of numerous proteins [49,50]. As a result, transient subclones acquiring excess, unnecessary, or even toxic chromosomal abnormality may be removed, even in cancer cells. Because the HMCLs utilized in this study were already fully established and transformed and possessed complicated chromosomal abnormalities [32], it is conceivable that subclones with further aneuploidy chromosomes were removed by cell competition with the dominant clone during the long-term culture. Given this scenario, further investigation using a disease model in which the development of de novo myeloma can be observed is needed to examine the function of BUB1. Finally, future study is expected to elucidate the association between BUB1 expression level and frequencies of gene mutations and/or gene dosage alterations.

In conclusion, this study revealed the possible association of BUB1 overexpression with CIN in MM. A further study is needed to determine the mechanism linking the clonal evolution of myeloma cells with BUB1 dysregulation, which may lead to new diagnostic and therapeutic approaches to limit myeloma progression.

## 4. Materials and Methods

### 4.1. Patient Samples

Bone marrow samples were obtained from patients with MGUS (*n* = 19), NDMM (*n* = 12), and RRMM (*n* = 27) between March 2014 and March 2018 at Kyoto Prefectural University of Medicine and Gunma University. MGUS/MM was diagnosed based on the International Myeloma Working Group 2014 criteria [51]. Bone marrow mononuclear cells were labeled with anti-CD138 MicroBeads, and CD138-positive plasma cells were isolated using a MACS separator (Miltenyi Biotec, San Diego, CA, USA). The purity of primary plasma cells after separation was 94.8 ± 2.4% (mean ± SD), as assessed by morphology [27]. The plasma cells of three healthy donors were used as control samples. This study was approved by the Institutional Review Boards of Kyoto Prefectural University of Medicine and Gunma University (Ethical code: RMBR-G-124-10). Patient samples were collected with informed consent in accordance with the Declaration of Helsinki.

### 4.2. Cell Lines and Reagents

Ten human myeloma-derived cell lines (HMCLs) were used in the study. AMO-1, NCI-H929, OPM2, and LP-1 were obtained from Deutsche Sammlung von Mikroorganisem und Zellkulturen (Braunschweig, Germany), and RPMI-8226 and IM9 were from American Type Culture Collection (Manassas, VA, USA). KMS-18, KMS-12-BM, KMS-28-PE, and KMS-34 are gifts from Dr. T. Ohtsuki (Kawasaki Medical School, Okayama, Japan). Most HMCLs were derived from an aggressive form of MM patients, such as with extramedullary disease or leukemic transformation (Table S1). Cells were maintained in RPMI-1640 medium containing 10% fetal calf serum, 2 mM L-glutamate, and 100 μg/mL penicillin/streptomycin at 37 °C in a fully humidified atmosphere of 5% CO_2_ in the air.

### 4.3. Quantitative RT-PCR

Total RNA extraction and quantitative RT-PCR (qRT-PCR) were performed, as described elsewhere [27,32,52,53]. Primer sequences for qRT-PCR were as follows: BUB1 FW: 5′-ACC ATT CCA CAA GCT TCC AGT G-3′, BUB1 RV: 5′-TGA AGG CAC CAC CAT GTT TTC C-3′, β-Actin (ACTB) FW: 5′-TTC TAC AAT GAG CTG CGT GTG G-3′, and ACTB RV: 5′-TGG GGT GTT GAA GGT CTC AAA C-3′.

### 4.4. Western Blotting

Western blotting was performed, as described previously [27,32,52,53], using rabbit anti-BUB1 (Abcam Cat#ab9000, Cambridge, UK) and mouse anti-ACTB (Sigma-Aldrich Cat# A2228, St. Louis, MO, USA) primary antibodies.

### 4.5. Lentiviral Preparation and Infection

BUB1 silencing was accomplished by stable expression of inhibitory short hairpin BUB1 RNAs (shRNAs). The target sequences for human BUB1 were 5′-GCA ACA ACA ATA CAG GTT ATT-3′ (shBUB1#1) and 5′-GCC TGA AGA TAC ATT CTA TTT-3′ (shBUB1#2). The sequences encoding shRNAs against BUB1 were cloned into lentivirus vector pLKO.1 puro (Addgene, Watertown, MA, USA). The empty vector was used as negative control (mock). Lentivirus was produced by transient co-transfection of HEK293T cells with a lentivirus vector, packaging plasmid pMDLg/pRRE, pRSV-Rev, and pMD2.G using PEI MAX (Polysciences, Warrington, PA, USA). Viral supernatant was collected 48 and 72 h after transfection and concentrated by ultracentrifugation. Infected cells were selected with 5 μg/mL puromycin beginning 48 h after infection and maintained under selection for 3–4 passages.

### 4.6. Immunofluorescence Microscopy

Cells were spun onto glass slides in a cytocentrifuge using Cytofuge 2 (Beckman Coulter, Brea, CA, USA) for 6 min at 1000 rpm, fixed with 4% paraformaldehyde, permeabilized with 0.2% Triton X-100, and blocked with 5% BSA in PBS for 20 min. Cells were incubated with anti-CENP-C antibody (MBL Cat#PD030, Nagoya, Japan) overnight at 4 °C and then incubated with goat anti-Guinea Pig IgG (H + L) secondary antibody, Alexa Fluor 546 (Invitrogen, Waltham, MA, USA), and 4′,6-diamidino-2-phenylindole (DAPI) for 1 h. Glass slides were then rinsed and mounted with ProLong Gold antifade reagent (Invitrogen) for imaging. A total of 100 cells were inspected using a fluorescence microscope BZ-X710 (Keyence Corp., Osaka, Japan).

### 4.7. Chromosomal Analysis

Conventional G-banding for metaphase spreads and fluorescence in situ hybridization (FISH) studies were performed, as described elsewhere [54]. Interphase FISH was performed using a Vysis CEP 3 (D3Z1) SpectrumOrange Probe and a Vysis CEP 7 (D7Z1) SpectrumGreen Probe (Abbott, Abbott Park, IL, USA) for the detection of centromeres of chromosome 3 (chr3) and chr7, respectively. Interphase cells were determined by the detection of non-mitotic nuclei stained with 4′,6-diamidino-2-phenylindole using BZ-X710.

### 4.8. Assays for Cell Proliferation and Cell Cycle

For the evaluation of cell proliferation, cells were seeded at 1 × 10^5^ cells/mL in a 24 well-plate, and cell number was counted by Trypan blue dye exclusion. For cell cycle analysis, cells were permeabilized and fixed with ice-cold 70% ethanol overnight, stained with propidium iodide (Sigma), and then subjected to flow cytometric analysis using a FACS Calibur (BD Biosciences, San Jose, CA, USA). Data obtained were analyzed using FLOWJO software Ver. X (Tomy Digital Biology, Tokyo, Japan) [48].

### 4.9. Colony-Formation Assay

KMS-18 cells were plated in Methocult H4230 (Stem Cell Technologies, Vancouver, BC, Canada) with 6% fetal bovine serum and 0.2% bovine serum albumin at 2000 cells/mL in triplicate dishes. RPMI-8226 cells were plated in the same medium at 250 cells/mL in triplicate dishes. After incubation for 14 days at 37 °C in a fully humidified atmosphere of 5% CO_2_ in the air, numbers of colonies were counted on an inverted microscope, and then colonies were fixed with methanol and stained with crystal violet dye.

### 4.10. Statistical Analysis

Data are expressed as mean ± SD of independent triplicate experiments. The significance of differences in the expression level of BUB1 was determined by one-way ANOVA with a Tukey multiple comparison test. Segregation errors, chromosomal number, cellular growth, and colony-forming ability were evaluated by the Student *t*-test. The distributions of whole chromosome numbers of 50 metaphase cells among HMLCs were compared by the F test. *p* < 0.05 was considered significant in all analyses.

## 5. Conclusions

In this study, we identified aberrant overexpression of BUB1 in myeloma cells, which associates with disease progression and aggressiveness. Our study suggested that enhanced BUB1 expression caused an increase in mitotic segregation errors and the resultant emergence of subclones with altered chromosome numbers, and thus, was involved in CIN in MM. Understanding the functional significance of BUB1 overexpression may lead to new diagnostic and therapeutic approaches to overcome myeloma progression.

## Supplementary Materials

The following are available online at https://www.mdpi.com/2072-6694/12/8/2206/s1, Supplementary information: Information about origins of human myeloma-derived cell lines utilized in this study, Table S1: Information about origins of human myeloma-derived cell lines utilized in this study, Figure S1: Original figures for whole Western blots of Figure 1c, Figure S2: Original figures for whole Western blots of Figure 2b, Figure S3: Association between cell proliferation rates and BUB1 protein levels in HMCLs, Figure S4: BUB1 knockdown reduces deviations from modal chromosome numbers.
