# Supplementary material for: Aberrant BUB1 Overexpression Promotes Mitotic Segregation Errors and Chromosomal Instability in Multiple Myeloma
Source: Cancers (Basel). 2020 Aug 6;12(8):2206. doi: 10.3390/cancers12082206 (PMC7464435; doi:10.3390/cancers12082206)
Supplement: Supplementary file 1 [file cancers-12-02206-s001.pdf]

Aberrant BUB1 overexpression promotes mitotic segregation errors and chromosomal instability in multiple myeloma

Yuto Fujibayashi, Reiko Isa, Daichi Nishiyama, Natsumi Sakamoto-Inada, Norichika Kawasumi, Junko Yamaguchi, Saeko Kuwahara-Ota, Yayoi Matsumura-Kimoto, Taku Tsukamoto, Yoshiaki Chinen, Yuji Shimura, Tsutomu Kobayashi, Shigeo Horiike, Masafumi Taniwaki, Hiroshi Handa and Junya Kuroda

Supplementary Table

Table S1. Information about origins of human myeloma-derived cell lines utilized in this study.

Stage	HMCL	Origin
Newly diagnosed MM	RPMI-8226	peripheral blood
Advanced MM	NCI-H929	pleural effusion
	KMS-34	
	KMS-28-PE	
	OPM-2	peripheral blood (plasma cell leukemia)
	KMS-18	
	LP-1	
	IM-9	bone marrow
	KMS-12-BM	
Newly diagnosed Plasmacytoma	AMO-1	ascites

AMO-1 was from ascites of patients with plasmacytoma, NCI-H929, KMS-34 and KMS-28-PE were from pleural effusion of advanced multiple myeloma (MM) patients, OPM2, KMS-18 and LP-1 were from peripheral blood of patients with plasma cell leukemia secondary to MM, RPMI-8226 was from peripheral blood of newly diagnosed MM patients, IM9 and KMS-12-BM were from bone marrow of advanced phase MM patients.

Supplementary Figures

Figure S1

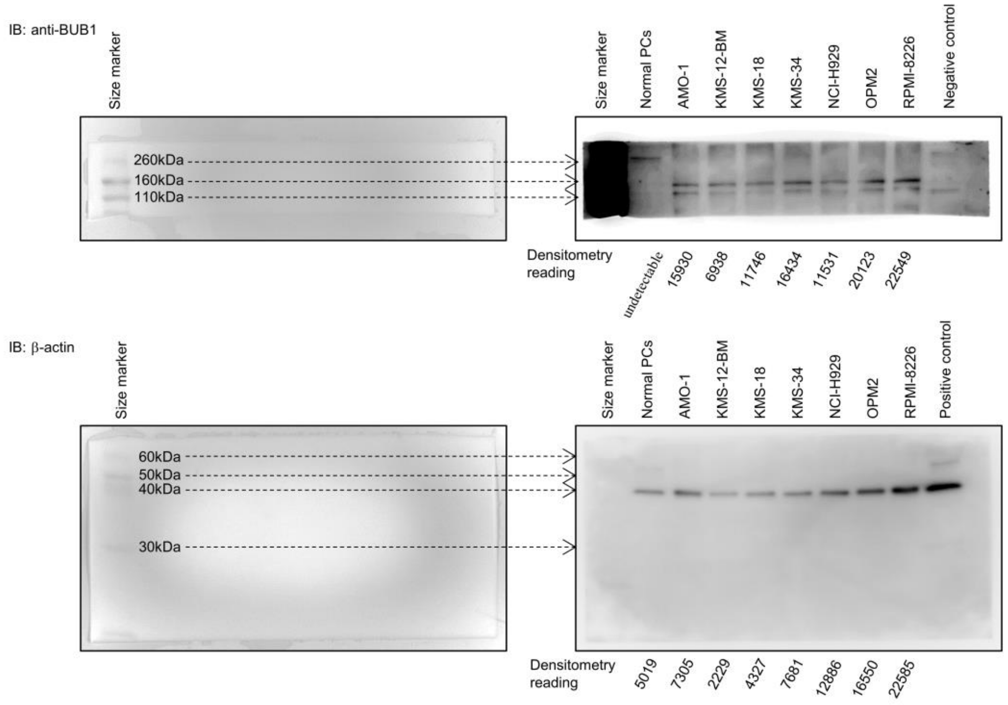

Figure S1. Original figures for whole Western blots of Figure 1c. The size marker (left) and the bands for target molecule (right) were scanned from the same membrane individually. Then, two photos were paralleled to confirm bands for target molecule by their molecular weight. IB: immunoblot.

Figure S2

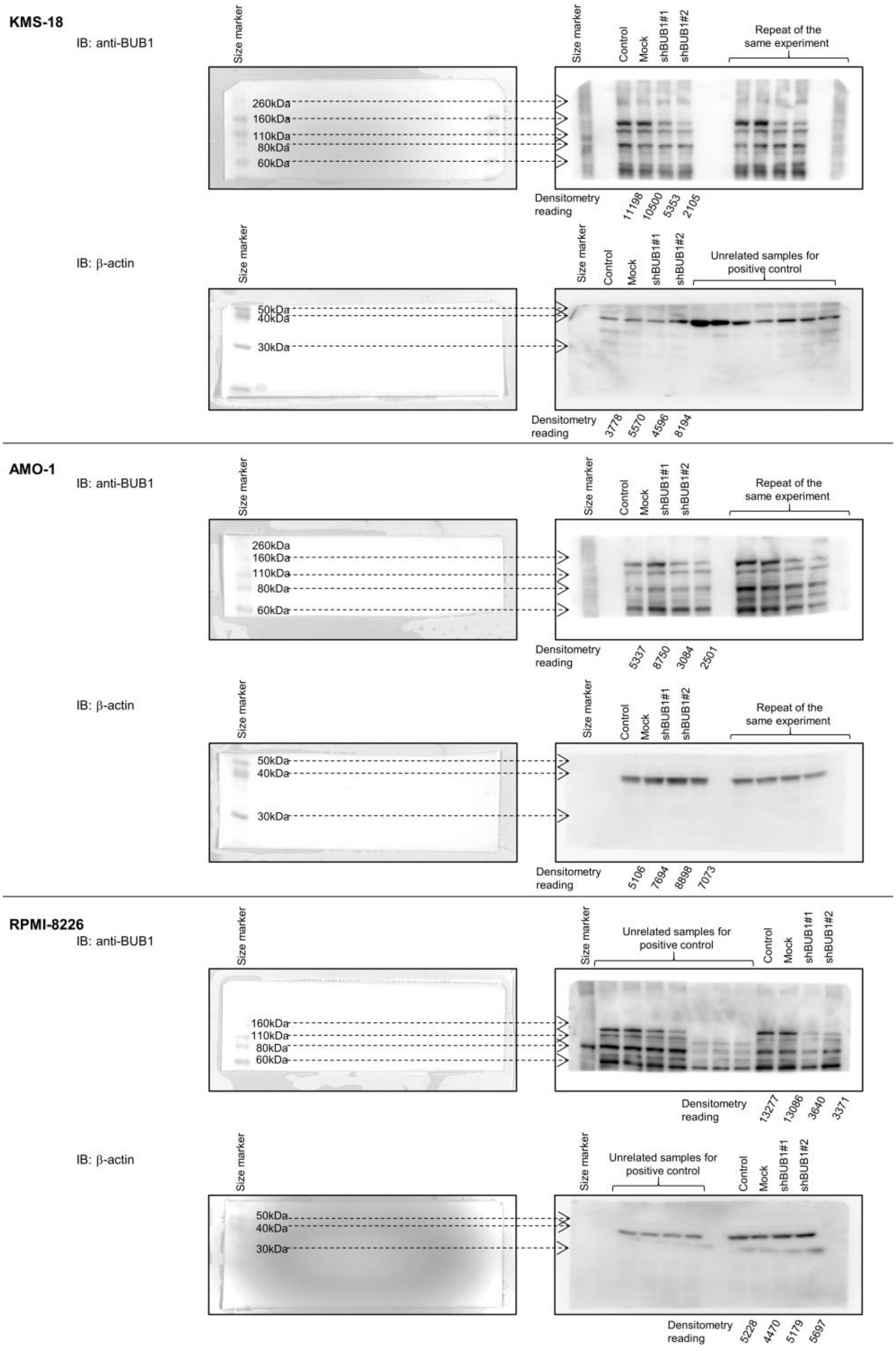

Figure S2. Original figures for whole Western blots of Figure 2b**.** The size marker (left) and the bands for target molecule (right) were scanned from the same membrane individually. Then, two photos were paralleled to confirm bands for target molecule by their molecular weight. IB: immunoblot.

Figure S3

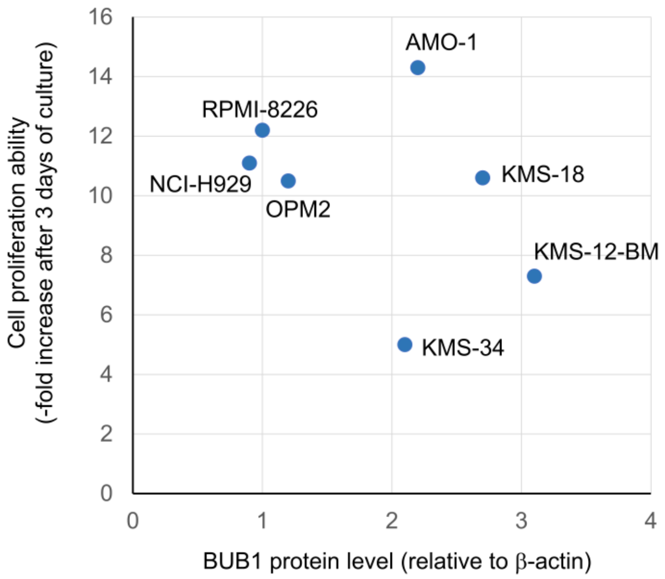

Figure S3. Association between cell proliferation rates and BUB1 protein levels in HMCLs. Fold changes in cell number over three days of culture are plotted on the vertical axis and BUB1 protein levels relative to b-actin are on the horizontal axis. Seven HMCLs were examined.

Figure S4

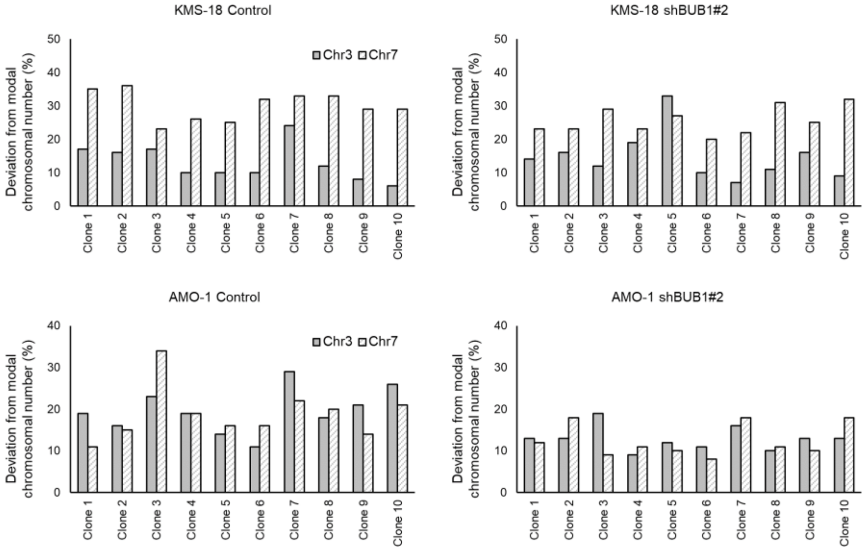

Figure S4. BUB1 knockdown reduces deviations from modal chromosome numbers. The percentage deviation from the modal chromosome number for the given chromosomes is shown. A total of 100 cells were counted in each clone.

© 2020 by the authors. Submitted for possible open access publication under the terms and conditions of the Creative Commons Attribution (CC BY) license (http://creativecommons.org/licenses/by/4.0/).
